# “People Like Us”: News Coverage of Food Assistance During the COVID-19 Pandemic

**DOI:** 10.1089/heq.2022.0001

**Published:** 2022-05-12

**Authors:** Pamela Mejia, Hina Mahmood, Sarah B. Perez-Sanz, Kim Garcia, Lori Dorfman

**Affiliations:** Berkeley Media Studies Group, Berkeley, California, USA.

**Keywords:** media, nutrition, public health, qualitative research

## Abstract

**Objective:**

To understand how the public discourse around food assistance and social responsibility evolved during the first year of the COVID-19 pandemic by analyzing news coverage.

**Methods:**

We conducted an ethnographic content analysis of news articles and photographs about food insecurity or food assistance published by U.S. newspapers and wire services between December 1, 2019, and November 30, 2020. We analyzed a random sample of 241 articles and 223 photographs to assess how they depicted food assistance programs, the program participants, and whether they included cues for deservingness.

**Results:**

Before the pandemic, news about food assistance was dominated by stories about abuse and fraud. Once COVID-19 began, news coverage contained cues known to engender beliefs about the deservingness of people receiving assistance. During the pandemic, news also highlighted misconceptions about food assistance programs, called for policy changes to reduce logistical barriers, and described the plight of families and other “people like us” in need of food assistance.

**Discussion:**

News coverage during the pandemic cued audience empathy, highlighted the logistical strains faced by food assistance programs, and elevated values of government accountability. The narrative about society's obligation to care for communities in need can be transferred to other safety net programs that protect the public's health.

**Health Equity Implications:**

As the pandemic evolves, public health leaders can maintain the narrative about the importance of food assistance and expand the characteristics of this narrative to challenge well-entrenched, but false, narratives about those who need help.

## Introduction

In the United States, debates about programs designed to ensure justice and provide economic inclusion for millions of people are rooted in contradictory ideals of rugged individualism and community interconnectedness. There is tension between the notion that health and well-being are shaped purely by personal choices and the belief that institutions, including government entities, also must act to protect the public's health.

The COVID-19 pandemic brought this tension into focus. For example, safety net programs, including the Supplemental Nutrition Assistance Program (SNAP, widely known as “food stamps”), have been politically charged for decades. Although the growth of SNAP has been hailed as a necessary expansion of our national safety net, it has also precipitated calls for stricter eligibility standards and stringent work requirements.

In late 2019, regulations were poised to take effect that would have made millions of people across the country ineligible for SNAP.^1,2^ These proposed policy restrictions were stalled by COVID-19, which brought food insecurity to the forefront of public debate.^3,4^ Ultimately, advocates won expansions that put food on the table for many already—and newly—hungry families.^5,6^

The public's perceptions of safety net program participants hinge on the concept of deservingness: people “determine if an individual deserves the benefits proposed by the policy [based on] a number of specific cues about the person, their problem, and their situation or broader context.”^7^ Deservingness cues—which include the actions of program participants, the public image of the participants, the design of benefit programs, ideological beliefs audience holds, and the audience's demographic characteristics—affect people's perceptions of *who* deserves support, and *how much* support they deserve to receive.^7^

For example, food assistance programs have been heavily politicized in part because there is stigma associated with receiving public assistance,^8^ which in turn affects how recipients are perceived. Perceptions of safety net program participants are also highly racialized; racial bias influences support for welfare programs.^9^

Analysis of news coverage of changes in SNAP before and after COVID-19 provides an opportunity to examine how safety net programs—and those who participate in them—are framed. News coverage both reflects public dialogue and sets the agenda for what topics the public and policymakers think about and how they think about them, including the policy solutions they support.^10–12^ In this study, we analyze news coverage of food assistance programs to determine whether and how deservingness cues were conveyed before and at the onset of the COVID-19 pandemic.

## Methods

**FIG. 1.**
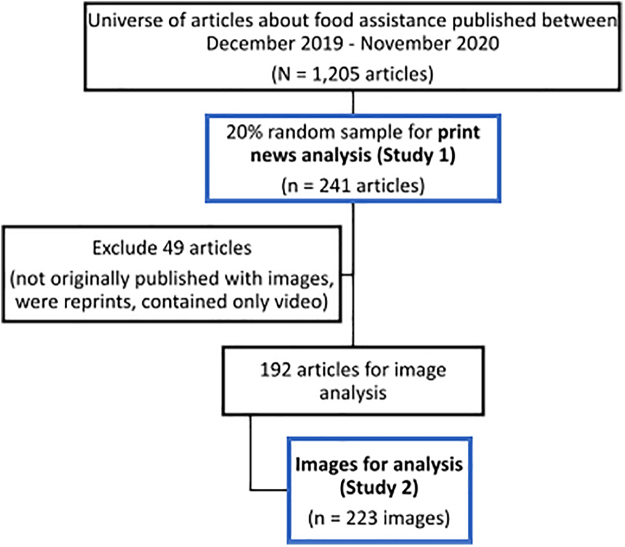
Sampling for Study 1 and Study 2.

We conducted paired ethnographic content analyses^13^ to examine news articles and associated photographs (Fig. 1).

### Data collection

#### Study 1

We searched the LexisNexis database and Gale OneFile News for national newspaper and wire articles published between December 1, 2019, and November 30, 2020, that included at least three mentions of terms such as food insecurity, food assistance, the Supplemental Nutrition Assistance Program (SNAP or food stamps), food banks or food pantries, or food waivers. We took a 20% random sample of articles from our total search results (*N*=1205) for in-depth coding (*N*=241).

Trained coders captured patterns in news coverage including mentions of (1) causes of food insecurity, (2) characteristics of people experiencing food insecurity, (3) solutions named to address food insecurity, and (4) actors or agencies explicitly assigned responsibility for addressing food insecurity. Before coding the final sample, coders held extensive conversations to achieve consensus on all coding variables.

#### Study 2

We searched Google for each article in our Study 1 sample to collect all images originally published with that article. We excluded any articles published without images or articles that contained only video (*n*=49). We analyzed 223 images associated with 192 articles.

Coding for Study 2 captured whether images depicted people, places, or objects. We examined images containing people to determine: the approximate age of the people pictured, whether the individuals appeared to be related, whether they presented as people of color or as white, whether people were depicted giving assistance or receiving assistance, and whether politicians or government buildings were pictured.

We used an iterative process to establish intercoder reliability and ensure agreement did not occur by chance. We first coded a randomly selected sample of photographs, then held consensus conversations to address and resolve differences in our coding. We then coded additional samples of photographs and held additional consensus discussions until we reached an acceptable level of agreement for all variables (Krippendorff's *α* ≥0.8).

### Analytical framework

We analyzed the data from both studies based on four of the five deservingness cues: the characteristics of participants, participants' public image, program design, and ideological beliefs about programs and participants.^7^

## Results

### News articles

Most stories about food assistance programs were published in the midwest (33%) and the south (33%); 19% were from outlets in the northeast and 11% from the west coast. Most (88%) were traditional news articles and 12% were opinion pieces.

**FIG. 2.**
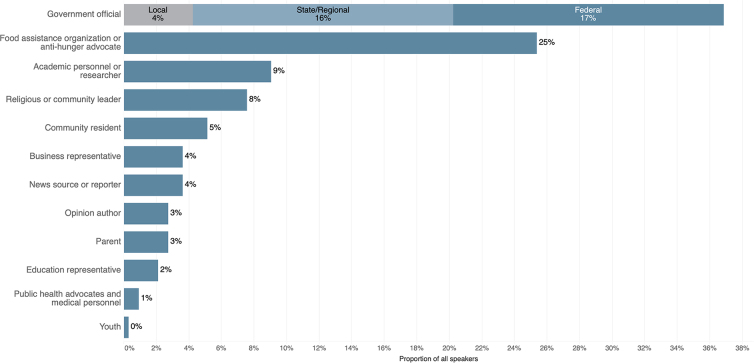
Speakers quoted in U.S. news coverage about food assistance, December 1, 2019 to November 30, 2020 (*N*=171 articles with direct quotations or attributions).

Representatives of local, state, and federal government dominated news about food assistance: 37% of articles included at least one quote from a government official. Antihunger advocates or representatives of food assistance organizations were quoted in 25% of articles. Speakers representing public health or medicine seldom appeared in the coverage (1%). Only 8% of the articles connected food access and health (Fig. 2).

More than half of articles (59%) described the COVID-19 pandemic as causing hunger by thrusting people into desperate situations. A typical article profiled a couple who sought assistance shortly after the onset of the pandemic since “both had recently lost their jobs … and were struggling to pay their bills.”^14^ Many people described applying for SNAP as a last resort, as when a parent decided to apply for the first time after he lost his jobs, calling it “a decision of survival.”^15^

Few articles linked structural inequities to poverty or food insecurity (13%): a rare example came from an opinion author who described poverty, and resultant hunger, as caused by “structural barriers such as poverty-level wages … or barriers to work such as caregiving responsibilities, disability or illness, or the stigma of a criminal record.”^16^

Almost one-third of the coverage (29%) referenced the structure of food assistance programs by, for example, describing logistical challenges like limited funding, as when a food bank director said, “we're running on a thread … and that thread is getting stretched.”^17^ Before and during the pandemic, people seeking food assistance described “red tape” that limited assistance. During the pandemic, many articles focused on the need to reduce these barriers by simplifying the application process, waiving work requirements, or enabling states to launch online purchasing through SNAP to “relieve some of the stress [recipients] face related to COVID-19 and improve access to good nutrition.”^18^

Families were mentioned as recipients of food assistance in 41% of articles; most of these references (79%) appeared in stories published after the onset of the pandemic in March of 2020. One volunteer for a neighborhood food relief program described participants as “the kids and the families we see on the soccer field and that my child sits next to in social studies class, the people who we are standing in line with in the grocery store.”^19^ Other articles emphasized the work ethic of food assistance recipients, as when a food stamp client who was seeking full time work said, “not everybody wants to be on government assistance … sometimes you just need some help …”^20^

Before the pandemic, news regularly reported on regulatory changes to SNAP aimed at preventing “food stamp fraud,” as when Brandon Lipps of USDA Food Nutrition and Consumer Service described the need to “restore integrity” to the program, continuing, “there's no asset test, so millionaires can legally come on the program.”^21^ No discussions of fraud appeared in articles published after March of 2020. Instead, news highlighted misconceptions about food assistance programs or called for systemic improvements, as when one opinion author asserted, “now that … so many people have seen the flaws of [the food assistance] paradigm firsthand—it's time we named the need for a shift.”^16^

**FIG. 3.**
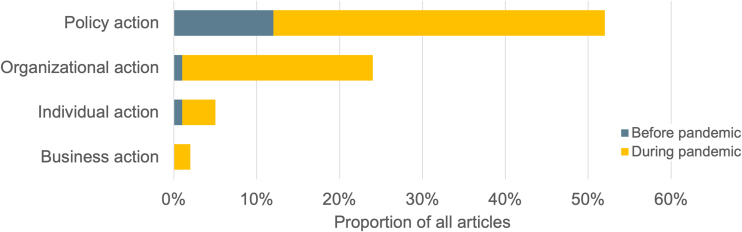
Solutions* to address hunger and food insecurity named in U.S. news coverage about food assistance, December 1, 2019 to November 30, 2020 (*N*=241 articles**). *Categories are not mutually exclusive. **Twenty-nine percent of the sample (69 articles) did not name a solution to hunger.

More than two-thirds of articles (72%) mentioned solutions to food insecurity, most of which (52%) were a state or federal policy. After the passage of the First Family Coronavirus Response Act, Congress member Rogers affirmed the need for emergency funding to “protect the health of employees and the sustainability of [business].”^22^ Articles that mentioned government policy solutions more than tripled during the pandemic (Fig. 3). Articles about solutions that involved private organizations (such as food banks expanding their hours) or individual actions (such as donating money or applying for SNAP benefits) followed a similar pattern though with fewer articles.

**FIG. 4.**
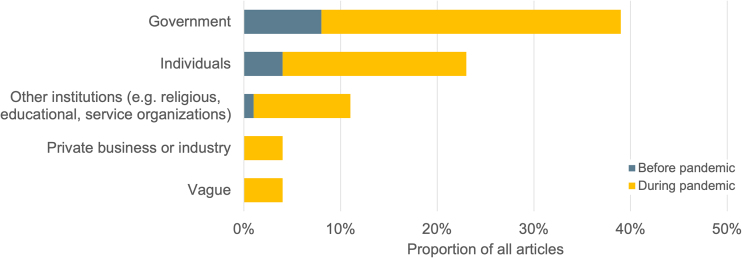
Groups* responsible for addressing hunger and food insecurity named in U.S. news coverage about food assistance, December 1, 2019 to November 30, 2020 (*N*=241 articles**). *Categories are not mutually exclusive. **Thirty-nine percent of the sample (94 articles) did not name any group as responsible for addressing hunger.

Explicit assertions of responsibility for enacting solutions for food insecurity most often named government actors (Fig. 4): 39% of all articles called on local, state, and federal government officials and agencies to act. Attorney General of North Carolina Josh Stein said, “many families are still struggling financially as a result of this pandemic,” and “urge[d] the federal government to ease these administrative burdens so that we can make sure North Carolinians can get the food assistance they need.”^23^ The number of articles that named government responsibility quadrupled during the pandemic. Similarly, mentions of the responsibility of businesses, community-based organizations, or individuals also appeared more often in news published during the pandemic.

### Photographs

In Study 2 we analyzed the 223 images associated with the articles from Study 1, of which 214 were photographs and 9 were graphics or drawings of objects (such as the coronavirus molecule or pictures of storefronts with signs indicating that Electronic Benefit Transfer [EBT] cards are accepted). Many images featured parking lots converted to food distribution sites (38%), or government buildings (21%), food banks (17%), and grocery stores (17%). Over one-third of the 223 images featured only inanimate objects such as food (35% of pictures of inanimate objects), EBT signage (17%), or cars waiting for food assistance (12%).

Most images (65%) featured people, usually unrelated groups of people (59%): only 6% of images of people pictured families. Most people profiled presented as adults (77%), 13% as elderly adults, and 11% as children; most featured people who presented as white (59%), compared with those who appeared to be people of color (39%). About half of photographs of people showed individuals providing food assistance, such as church groups distributing food in parking lots. People pictured receiving food assistance were nearly evenly split between those who presented as white (52%) and those who presented as people of color (48%).

Before the onset of the pandemic, five articles included mugshots of people accused or convicted of cheating food assistance programs, all of whom presented as people of color. Articles published after the onset of the pandemic did not include mugshots.

## Discussion

News about food assistance programs shifted with the onset of COVID-19. During the pandemic, news coverage far more often evoked government responsibility for addressing hunger and referenced policy solutions at the federal, state, and local levels of government. News during the pandemic highlighted the need to reduce barriers to food assistance.

Before the pandemic, news about food assistance was dominated by stories that evoked abuse and fraud, along with the occasional mugshot of people accused of fraud. During the pandemic, no articles or photographs of people accused of fraud were published. News during the pandemic more often described (though did not picture) families who participated in food assistance programs or included language that evoked empathy for recipients, framing them as “people like us” (Table 1).

**Table 1.** Deservingness Cues in U.S. News Coverage About Food Assistance Before and During the Onset of COVID-19, December 1, 2019 to November 30, 2020

Deservingness cue	News characteristics before COVID-19	News characteristics at the onset of COVID-19
Participant behaviors: information about participants' behaviors influences beliefs about deservingness.	News rarely referenced families as recipients of food assistance.	News included many references to families as food assistance recipients.
Public image: Stigma, prejudice, or stereotypes about the participant groups affect deservingness judgments.	News evoked abuse of food assistance programs.	News about fraud and abuse fell off markedly.
Program design: The structure of benefit programs influences deservingness perceptions.	News focused on logistical barriers to food assistance.	News focused on efforts to simplify processes to access food and reduce barriers.
Ideological beliefs: Audience's values related to social order and the role of government affect deservingness judgments.	News contained few articles about policy solutions at the federal, state, or local levels of government.	Almost half of news articles named policy solutions at the federal, state, or local levels of government.

Four cues about deservingness regularly appeared in news about food assistance published before and during the first year of the pandemic:

### Individual behavior: Food assistance recipients were portrayed as desperate and “suddenly hungry” because of COVID-19

People working to get out of their situation are seen as deserving because their actions demonstrate personal responsibility, compliance, and a strong work ethic.^7^ Articles about food assistance characterized individuals who receive benefits as hardworking and grateful for support amid desperate circumstances. Many articles positioned the pandemic as a catastrophe beyond any individual's control that thrust previously self-reliant people into extreme situations and forced them to seek support.

### Public image: After the onset of COVID-19, program participants were portrayed empathetically as “people like us” in news coverage

News coverage that highlights similarities between program participants and news audience helps audience view participants more sympathetically and judge them deserving of support.^7^ Coverage published after the onset of the pandemic focused on the needs of relatable sympathetic groups, including families, experiencing hunger.

Beliefs about specific racial groups rooted in white supremacy and systemic racism likely also influence the public image of participants and audience's judgments about their deservingness. Stories about food assistance that over-represent people of color could perpetuate stereotypes that hunger—and poverty more broadly—is primarily experienced by communities of color, although white people make up a large proportion of participants (37%) in food assistance programs such as SNAP.^24^ During the pandemic, there were almost equal number of photographs showing white people receiving assistance as people of color, which is a close representation of the racial makeup of SNAP participants but a reversal from previous depictions that over-represented people of color.^25^

### Institutional design: COVID-19 transformed how programs were portrayed and elevated institutional stress

Deservingness judgments are also influenced by whether social programs are described as vulnerable to misuse or as effective and efficient. Stories about food assistance during the pandemic highlighted logistical stresses and the need to address them promptly, at times describing policy changes that relieved cumbersome regulations and other types of bureaucratic “red tape” to facilitate more immediate and comprehensive assistance for more people.

Representatives of food assistance organizations frequently spoke in news coverage (see Fig. 2), and named the surge in demand, depletion of operational resources, and challenges they faced. Photographs of lines of cars waiting in parking lots at busy food pantries visually confirmed heightened demand and stretched resources. The deservingness rubric suggests that these depictions of contextual factors could engender empathy and support.

### Ideological beliefs: News coverage during COVID-19 emphasized values of government accountability and personal charity

Political ideology, moral values, and core belief systems influence deservingness judgments. Ideas around social hierarchy and the importance of meritocracy can lower support for government assistance.^7^ News about food insecurity and food assistance programs after the onset of the pandemic differed from typical news, which tends to focus on problems rather than solutions,^26^ because it regularly named solutions (Fig. 3). The emphasis on policy solutions was reinforced by images that featured both federal and state government officials and buildings.

Values of charity and personal giving were reflected in the images that accompanied articles, many of which featured church groups distributing food in parking lots or makeshift distribution sites. Ideological beliefs about the importance of personal giving and the need for government action sometimes came into conflict in stories about food assistance, particularly in articles that celebrated the actions of private organizations and donors to address hunger, though they also noted that charity could not replace government action.^27^

Overall, news during the beginning of the COVID-19 pandemic evoked deservingness by painting an inclusive picture of desperate recipients of food assistance, illustrating the strains faced by food assistance programs, elevating solutions that reduced “red tape” and expanded access, and highlighting values of personal charity and government accountability.

Our research has several limitations. Because we did not test audience reactions to the news, we could not evaluate how characteristics of news audience affected their deservingness judgments.^7^ Our assessment of photographs is limited to news articles with affiliated images and we could not locate images associated with one-fifth of the articles in our analysis. We did not assess video, which may have conveyed additional cues about race, class, and gender.

## Health Equity Implications

This study is, to our knowledge, the first to explore how deservingness cues are transmitted in news coverage about food assistance programs. We found a shifting frame that could be leveraged to maintain an ongoing narrative about food assistance that elevates the dignity of program participants, centers government action, and makes the case for continuing to streamline programs and reduce logistical burdens for both participants and administrators.

In contrast to traditional news frames of individualism and “bootstrapping,”^26^ responsibility for addressing food insecurity during the pandemic was clearly assigned to the federal government. This helps make the case for increasing access to food assistance, highlighting benefits for families and communities, and reducing accusations of fraud and stigma associated with their use, all of which support the long-term expansion of safety net programs that benefit the public's health.^28^

The COVID-19 narrative around food assistance portrays a more empathetic and broad depiction of program participants. The news visually depicted a wide range of people receiving food assistance during the pandemic and framed recipients as hard working and relatable, characteristics that are also embodied by participants absent a pandemic. Cumulatively, these cues could increase audience's ability to empathize with program participants. If people experiencing hunger see people they identify with depicted empathetically in news coverage, that may also reduce the stigma and shame associated with receiving government support^29^ that keeps eligible families from enrolling.^30^

Prior studies of food assistance programs have found little evidence of equity language in news coverage, even though such safety net programs are designed to address the health consequences of inequities.^31^ News coverage at the onset of COVID-19 demonstrates an equity-focused narrative that highlights the benefits of government assistance without stigmatizing participants in part because the pandemic thrust people into a situation out of their control. However, the need these “suddenly hungry” families felt is akin to what many people experienced before the pandemic.

Moving forward, the entrenched harsh individualistic framing of safety net programs, and less empathetic cues about people who use them,^7^ could re-emerge. We can resist the return to “normal,” and instead challenge abiding, but false, narratives about those in need of assistance from our nation's safety nets and insist on sustained government action to ensure equitable access to food whenever people need it.

## Abbreviations Used

EBT: Electronic Benefit Transfer
SNAP: Supplemental Nutrition Assistance Program

## References

[1] Wheaton L. Estimated Effect of Recent Proposed Changes to SNAP Regulations. Washington, DC: Urban Institute, 2019, p. 26.

[2] Rosenbaum D, Dean S, Bolen E, et al. President's Budget Would Cut Food Assistance for Millions and Radically Restructure SNAP. Washington, DC: Center on Budget and Policy Priorities, 2018, p. 15.

[3] Schanzenbach DW. Not Enough to Eat: COVID-19 Deepens America's Hunger Crisis. Washington, DC: Food Research & Action Center, 2020.

[4] *The Impact of the Coronavirus on Food Insecurity in 2020*. Chicago, IL: Feeding America, 2020.

[5] Godoy M. Judge blocks rule that would have kicked 700,000 people off SNAP. NPR. 2020. Available at https://www.npr.org/sections/thesalt/2020/03/14/815748914/judge-blocks-rule-that-would-have-kicked-700-000-people-off-snap Accessed November 10, 2021.

[6] Guardia L. Food Research & Action Center Commends the House for Passing the Families First Coronavirus Response Act. Food Research & Action Center. 2020. Available at https://frac.org/news/food-research-action-center-commends-the-house-for-passing-the-families-first-coronavirus-response-act Accessed November 10, 2021.

[7] Foster-Fishman P. White Paper Prepared for Robert Wood Johnson Foundation: Understanding Deservingness and Its Impact on Pro-Social Policy Support. Princeton, NJ: Robert Wood Johnson Foundation. 2020.

[8] Gaines-Turner T, Simmons JC, Chilton M. Recommendations from SNAP participants to improve wages and end stigma. Am J Public Health. 2019;109:1664–1667. 31622134 10.2105/AJPH.2019.305362 PMC6836769

[9] Brown-Iannuzzi JL, Dotsch R, Cooley E, et al. The relationship between mental representations of welfare recipients and attitudes toward welfare. Psychol Sci 2017;28:92–103. 27879320 10.1177/0956797616674999

[10] Dearing JW, Rogers EM. Agenda-Setting. Thousand Oaks, CA: Sage, 1996.

[11] Iyengar S. Is Anyone Responsible? How Television Frames Political Issues. Chicago, IL: The University of Chicago Press, 1991.

[12] McCombs M, Shaw D. The agenda-setting function of mass media. Public Opin Q. 1972;36:176–187.

[13] Altheide DL. Reflections: ethnographic content analysis. Qual Sociol. 1987;10:65–77.

[14] Richler J. Food insecurity during pandemic a real concern for Jews. South Florida Sun-Sentinel. 2020. Available at https://www.sun-sentinel.com/florida-jewish-journal/fl-jj-food-shortage-concern-jews-hit-hard-pandemic-20200512-qg2k4ocscbaizfevm2xti5qni4-story.html Accessed November 10, 2021.

[15] Chang J. Coronavirus Crisis Leads to Surge in Texas Food Stamp Applications. Austin, Texas: Austin American-Statesman. 2020.

[16] Vallas R. Republicans wrapped the safety net in red tape. Now we're all suffering. Washington Post. 2020. Available at https://www.washingtonpost.com/outlook/2020/04/15/republicans-harder-access-safety-net/ Accessed November 10, 2021.

[17] Burris A. “Everyone is hurting”: indiana food bank director says group will soon be out of money. IndyStar. 2020. Available at https://www.indystar.com/story/money/2020/05/20/indianapolis-midwest-food-bank-run-out-money/5218064002/ Accessed November 10, 2021.

[18] Roth C. Missouri food stamp users can buy online at 2 retailers. Rolla Daily News. May 16, 2020.

[19] Corr J. Neighbors ensure a Thanksgiving feast for all. Herald Community Newspapers. 2020. Available at https://www.liherald.com/stories/neighbors-ensure-a-thanksgiving-feast-for-all,128779 Accessed November 10, 2021.

[20] Fadulu L. Cities Prepare for the Worst as Trump's Food Stamp Cuts Near. The New York Times. 2020. Available at https://www.nytimes.com/2020/01/25/us/politics/trumps-food-stamp-cuts.html Accessed November 10, 2021.

[21] Nexstar Media Inc. USDA says food stamp regulations will prevent misuse, Trump admin sued over ‘assault on poor people.’ 2020. Available at https://fox4kc.com/news/usda-says-food-stamp-regulations-will-prevent-misuse-trump-admin-sued-over-assault-on-poor-people/ Accessed November 10, 2021.

[22] Neal J. Rogers, McConnell support relief efforts for Coronavirus fallout. Commonwealth Journal. 2020. Available at https://www.somerset-kentucky.com/covid-19/rogers-mcconnell-support-relief-efforts-for-coronavirus-fallout/article_bc0c3e66-6631-11ea-afb8-e350f5ebd8c7.html Accessed December 2, 2021.

[23] Daniel N. North Carolina AG joins other states seeking continued waivers for food stamps. The Center Square. 2020. Available at https://www.thecentersquare.com/north_carolina/north-carolina-ag-joins-other-states-seeking-continued-waivers-for-food-stamps/article_a9795320-f9f1-11ea-9bac-1bea5a317133.html Accessed November 10, 2021.

[24] Cronquist K. Characteristics of Supplemental Nutrition Assistance Program Households: Fiscal Year 2019. Alexandria, Virginia: United States Department of Agriculture, Food and Nutrition Service, Office of Policy Support, 2021, p. 162.

[25] Gilen M. Race and poverty in America: Public misperceptions and the American News Media. Public Opin Q. 1996;60:515–541.

[26] Dorfman L, Wallack L, Woodruff K. More than a message: Framing public health advocacy to change corporate practices. Health Educ Behav. 2005;32:320–336. 15851542 10.1177/1090198105275046

[27] O'Malley Bunder K. Food Finders plea: Increase benefits to fight hunger. Journal & Courier, 2000. https://www.jconline.com/story/news/opinion/letters/2020/05/07/food-finders-plea-increase-benefits-fight-hunger-next-coronavirus-stimulus-bill/3093533001/

[28] United States Department of Agriculture. USDA modernizes the Thrifty Food Plan, Updates SNAP Benefits. 2021. Available at https://www.usda.gov/media/press-releases/2021/08/16/usda-modernizes-thrifty-food-plan-updates-snap-benefits Accessed November 12, 2021.

[29] Swales S, May C, Nuxoll M, et al. Neoliberalism, guilt, shame and stigma: A Lacanian discourse analysis of food insecurity. J Community Appl Soc Psychol. 2020;30:673–687.

[30] Fang D, Thomsen MR, Nayga RM. The association between food insecurity and mental health during the COVID-19 pandemic. BMC Public Health. 2021;21:607. 33781232 10.1186/s12889-021-10631-0 PMC8006138

[31] Winett L, Dorman L, Yoshino L, et al. Equity arguments in news reporting on school nutrition policy. Health Equity. 2:117–121. 30283857 10.1089/heq.2017.0061 PMC6071905

